# A chromosomal analysis of four species of Chilean Chrysomelinae (Coleoptera, Chrysomelidae)

**DOI:** 10.3897/CompCytogen.v6i4.3519

**Published:** 2012-10-05

**Authors:** Eduard Petitpierre, Mario Elgueta

**Affiliations:** 1 Dept. Biologia, Universitat de les Illes Balears, 07122 Palma de Mallorca, Spain; 2 Área Entomología, Museo Nacional de Historia Natural, Casilla 787, Santiago, (Correo Central), Chile

**Keywords:** Coleoptera, Chrysomelidae, Chrysomelinae, karyotypes, cytotaxonomy

## Abstract

Four species of Chilean leaf beetles in the subfamily Chrysomelinae have been cytogenetically analyzed, *Blaptea elguetai* Petitpierre, 2011, *Henicotherus porteri* Bréthes, 1929 and *Jolivetia obscura* (Philippi, 1864) show 2n = 28 chromosomes and a 13 + Xy_p_ male meioformula, and *Pataya nitida* (Philippi, 1864) has the highest number of 2n = 38 chromosomes. The karyotype of *Henicotherus porteri* is made of mostly small meta/submetacentric chromosomes, and that of *Jolivetia obscura* displays striking procentric blocks of heterochromatin at pachytene autosomic bivalents using conventional staining. These findings are discussed in relation to previous cytogenetic data and current taxonomy of the subfamily.

## Introduction

The subfamily Chrysomelinae is a group of mostly quite large or medium sized leaf beetles mainly distributed in cool and temperate regions of the world, which are composed of 133 genera (Daccordi 1994), and nearly 3000 species worldwide (Farrell 1998; Reid et al. 2009)

From the cytogenetic standpoints, this subfamily is relatively well-known since nearly 260 taxa and chromosomal races in 38 genera have been surveyed to date (Petitpierre 2011a). In a previous cytogenetic study, we analyzed three Chilean species of Chrysomelinae (Petitpierre and Elgueta 2006), belonging to three of the ten genera so far found in the country (Daccordi 1994). We have here enlarged this research with four additional species and genera from Chile, of which three, *Henicotherus* Bréthes, 1929,* Jolivetia* Bechyné, 1946 and *Pataya* Bechyné, 1946, are endemics for this geographic subregion in the Neotropics (Daccordi 1994), and the fourth, *Blaptea* Weise, 1915, has only one other species, in Colombia and Brazil (Daccordi 1994, Petitpierre 2011b).

## Material and methods

The checked species and their origins are reported in Table 1.

**Table 1.** Chromosomally checked species and their Chilean geographical sources.

Blaptea elguetai Petitpierre, 2011	Isla Negra, prov. San Antonio, Reg. Valparaíso
Henicotherus porteri Bréthes, 1929	Mincha 2 km W, prov. Choapa, Reg. Coquimbo
Jolivetia obscura (Philippi, 1864)	Isla Negra, prov. San Antonio, Reg. Valparaíso
Pataya nitida (Philippi, 1864)	Isla Negra, prov. San Antonio, Reg. Valparaíso

The surveyed individuals of *Blaptea elguetai*, *Jolivetia obscura* and *Pataya nitida* were caught by sweeping on their host plants *Tropaeolum brachyceras* Hook. et Arn., 1830 (Tropaeolaceae), *Aristeguietia salvia* (Colla) R.M. King et H. Rob., 1975 (Asteraceae) and *Ageratina glechonophylla* (Less.) R.M. King et H. Rob., 1970 (Asteraceae), in October 2009, October 2006 and November 2007, respectively, and those of *Henicotherus porteri* were caught by hand under stones in October 2009. At least two individuals from each species have been cytogenetically studied.

The chromosome analyses were only performed on male living individuals brought from Chile to our laboratory in Palma de Mallorca (Spain), where they were killed with ethyl acetate. Then, the procedure to get the conventional staining preparations was the same used before in our previous paper (Petitpierre and Elgueta 2006). Finally, we obtained micrographs by a ZEISS AXIOSKOP photomicroscope and subsequently enlarged them for printing at X1500.

## Results

### Tribe Chrysomelini. Subtribe Entomoscelina

**Figures 1A–C.**
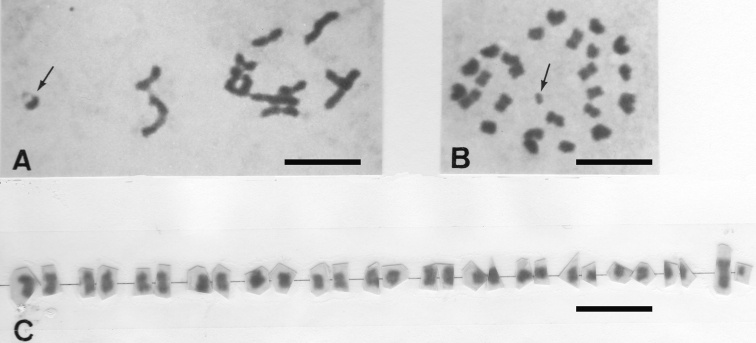
**A** *Blaptea elguetai* metaphase I showing 13 + Xy_p_, the Xy_p_ is arrowed **B** *Henicotherus porteri* spermatogonial metaphase with 2n = 28, the y-chromosome is arrowed.**C** karyogram showing small meta/submetacentric chromosome pairs, the medium-sized X and the smallest y-chromosome are in the extreme right. Bar = 10 µm

*Blaptea elguetai* has 2n = 28 chromosomes and a 13 + Xy_p_ male meioformula of medium and small autosomal bivalents plus the Xy_p_ “parachute” sex-chromosome system where most of these autosomic bivalents are rod-shaped (Fig. 1A).

*Henicotherus porteri* has also 2n = 28 chomosomes at spermatogonial metaphases (Fig. 1B), from which a karyogram has been obtained, made of medium and small metacentrics of gradually decreasing sizes, including the largest X-chromosome and the smallest y-chromosome elements (Fig. 1C). Confirming what was expected, the metaphases I comprise 13 autosomic bivalents and the Xy_p_ sex-chromosome system (not shown).

### Tribe Chrysomelini. Subribe Chrysomelina

**Figures 2A–C.**
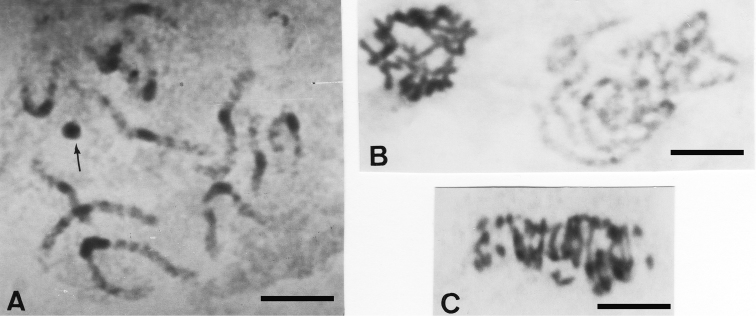
**A** *Jolivetia obscura* pachytene showing 13 + Xy_p_ with striking procentric heterochromatic bands in the autosome bivalents, and the presumed Xy_p_ arrowed **B** *Pataya nitida* spermatogonial metaphase (left) and pachytene (right), some autosome bivalents show small procentric bands of heterochromatin **C** anaphase I showing 2n = 38 chromosomes. Bar = 10 µm

*Jolivetia obscura* displays 2n = 28 chromosomes as in the two previous species, from pachytene meiotic cells where 14 bivalents are distinguishable and among them the Xy_p_ sex-chromosome system. Each of the 13 pachytene autosomal bivalents show a remarkable band of procentric heterocromatin, and the presumed Xy_p_ sex-chromosome system appears as a strongly heterochromatic round bulk under the conventional staining technique (Fig. 2A).

Conversely, *Pataya nitida* displays a higher diploid number of 19 pachytene bivalents, a few of which having heterochromatic bands (Fig. 2B), and 2n = 38 small chromosomes at spermatogonial anaphase (Fig. 2C).

## Discussion

The diploid number of chromosomes and male sex-chromosome system of *Blaptea elguetai* 2n = 28 (Xy_p_) agrees with our findings in *Microtheca ochroloma* Stål, 1860 (Petitpierre 1988), *Blaptea* and *Microtheca* Stål, 1860, both American genera, are closely related taxa within the subtribe Entomoscelina (Daccordi 1994). *Henicotherus porteri*, also belonging to the same subtribe Entomoscelina as the former (Daccordi 1994), shares again a 2n = 28 (Xy_p_) diploid number and male sex-chromosome system, and its karyotype is made up of meta/submetacentric chromosomes of small size mostly. These meta/submetacentric chromosome shapes are the prevalent elements in beetle karyotypes (Smith and Virkki 1978; Virkki 1984), and more particularly, in the leaf beetles of the subfamily Chrysomelinae too (Petitpierre 2011a).

Among the different subtribes of Chrysomelinae (Daccordi 1994), the Entomoscelina have been scarcely surveyed from cytogenetic standpoints, with only seven checked species (Barabás and Bezo 1978; Petitpierre 1988; Petitpierre and Grobbelaar 2004), in five genera including the two present ones, among the total of 27 genera described to date (Daccordi 1994). However, it might seem that this subtribe is rather conservative in chromosome number and sex-chromosome system because five species have 2n = 28 (Xy_p_) and two 2n = 26 (Xy_p_), contrary to most other subtribes of Chrysomelinae, which exhibit a wide range of haploid chromosome numbers, namely from 9 to 22 in Timarchina, 10 to 25 in Chrysolinina, and 6 to 18 in Doryphorina (Petitpierre 2011a).

As reported above, *Jolivetia obscura* and *Pataya nitida* are classified in a different subtribe, Chrysomelina, than the two previous species (Daccordi 1994), and they have 2n = 28 (Xy_p_) and 2n = 38 chromosomes, respectively. Among the 35 chromosomally studied species belonging to 12 genera in this subtribe, there is again a rather wide range of haploid numbers from 12 to 19, but with a clear modal value at n = 17 (65.7%) (Petitpierre 2011a). Therefore, *Pataya nitida* displays the highest so far found number and *Jolivetia obscura* one of the lowers within subtribe Chrysomelina. It is also remarkable, that even though both species, *Jolivetia obscura* and *Pataya nitida*, are taxonomically and morphologically related (Daccordi 1994), feeding on Asteraceae host plants as mentioned above, they are characterized with so diverse chromosome numbers.

The procentric bands of heterochromatin found in pachytene autosomal bivalents of *Jolivetia obscura* and in some of those of *Pataya nitida*, using conventional staining, are common feature in beetle chromosomes, as it has been recently demonstrated in several families of Coleoptera including Chrysomelidae by C-banding techniques (Rozek et al. 2004; Karagyan et al. 2012).

The sex-chromosome system found in our sampled species of Chilean chrysomelines was the parachute-type Xy_p_, except in *Pataya nitida* which has not been identified. Thus, they agree with those found in the three previously analysed species of Chilean chrysomelines (Petitpierre and Elgueta 2006), and follow the prevalent rule in the subfamily Chrysomelinae, where almost 80% of the nearly 260 examined taxa display this sex-chromosome system (Petitpierre 2011a), as well as it also occurs in most beetles of the suborder Polyphaga (Smith and Virkki 1978; Virkki 1984; Dutrillaux and Dutrillaux 2009).
